# The Role of Fibroblasts in Skin Homeostasis and Repair

**DOI:** 10.3390/biomedicines12071586

**Published:** 2024-07-17

**Authors:** Federica Boraldi, Francesco Demetrio Lofaro, Susanna Bonacorsi, Alessia Mazzilli, Maria Garcia-Fernandez, Daniela Quaglino

**Affiliations:** 1 Department of Life Science, University of Modena and Reggio Emilia, 41125 Modena, Italy; francescodemetrio.lofaro@unimore.it (F.D.L.); susanna.bonacorsi@unimore.it (S.B.); alessia.mazzilli@unimore.it (A.M.); 2 Department of Human Physiology, Institute of Biomedical Investigation (IBIMA), University of Málaga, 29010 Málaga, Spain; igf@uma.es

**Keywords:** fibroblast, extracellular matrix, mechanobiology, aging, skin repair, disease, proteomics

## Abstract

Fibroblasts are typical mesenchymal cells widely distributed throughout the human body where they (1) synthesise and maintain the extracellular matrix, ensuring the structural role of soft connective tissues; (2) secrete cytokines and growth factors; (3) communicate with each other and with other cell types, acting as signalling source for stem cell niches; and (4) are involved in tissue remodelling, wound healing, fibrosis, and cancer. This review focuses on the developmental heterogeneity of dermal fibroblasts, on their ability to sense changes in biomechanical properties of the surrounding extracellular matrix, and on their role in aging, in skin repair, in pathologic conditions and in tumour development. Moreover, we describe the use of fibroblasts in different models (e.g., in vivo animal models and in vitro systems from 2D to 6D cultures) for tissue bioengineering and the informative potential of high-throughput assays for the study of fibroblasts under different disease contexts for personalized healthcare and regenerative medicine applications.

## 1. Introduction

In 1858, Virchow firstly described “spindle-shaped cells of the connective tissue” [1], but it was Ziegler who, 40 years later, used the term “fibroblast” to describe the cells depositing new connective tissue in wounds [2].

Fibroblasts are typical mesenchymal cells widely distributed throughout the human body, where they (1) synthesise and maintain the extracellular matrix (ECM), ensuring the structural role of soft connective tissues; (2) secrete cytokines and growth factors; (3) communicate with each other and with other cell types, acting as signalling source for stem cell niches; and (4) are involved in tissue remodelling, fibrosis, wound healing, and cancer [3]. Indeed, from the functional point of view and with a partially overlapping phenotype, it is possible to recognize fibrosis-associated fibroblasts (FAF), wound healing-associated fibroblasts (WAF), cancer-associated fibroblasts (CAF) and aging-associated fibroblasts (AAF). Therefore, although fibroblasts isolated from different tissues exhibit similarities in appearance, suggesting that they can be considered interchangeable, they indeed unveil differences depending on the morpho-functional specificities of the environment where they are located and on their origin [4].

Fibroblasts are considered one of the most widely used model systems to understand comparative physiology, to investigate pathologic conditions through reductionist approaches, and for applications in biomedicine since they are easily isolated from tissues and can be grown in culture on artificial surfaces such as glass and plastic or on natural and/or bioengineered materials [5]. Similarly to the spontaneously immortalized 3T3 fibroblast cell line, originally derived from mouse embryo [4], fibroblasts are widely used in basic cellular and molecular biology studies since they retain excellent growth capabilities for several passages and exhibit high environmental plasticity, offering the possibility to be reprogrammed to become pluripotent stem cells for a wide spectrum of applications in regenerative medicine [6,7].

## 2. Origin of Fibroblasts and Their Heterogeneity

**Figure 1:**
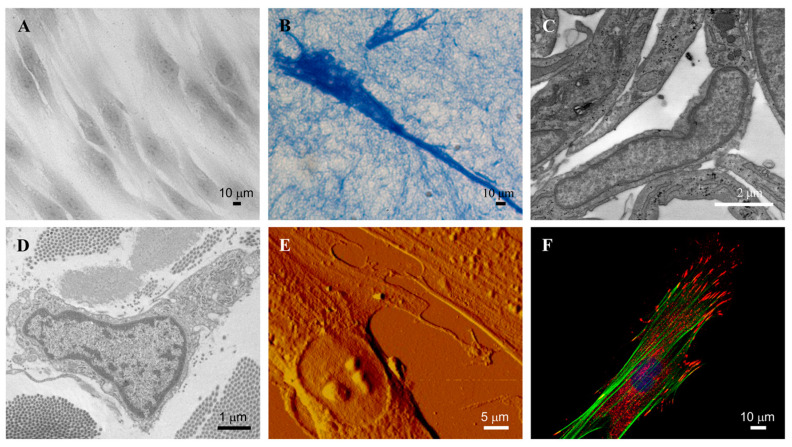
Representative images of dermal fibroblasts. Fibroblasts in 2D (**A**) and 3D (**B**) culture observed by light microscopy. Fibroblast in culture (**C**) and in healthy skin (**D**), observed by transmission electron microscopy. Fibroblast in culture observed by atomic force microscopy (**E**) and by confocal microscopy (**F**). Images are from authors’ laboratory.

Despite their conventional morphological description as spindle- or stellate-shaped cells with an oval nucleus and a distinct endoplasmic reticulum (Figure 1), it is now well known that fibroblasts have distinctive embryological origin, differently interact with other cells and play multiple roles in many pathologic contexts [8].

**Figure 2:**
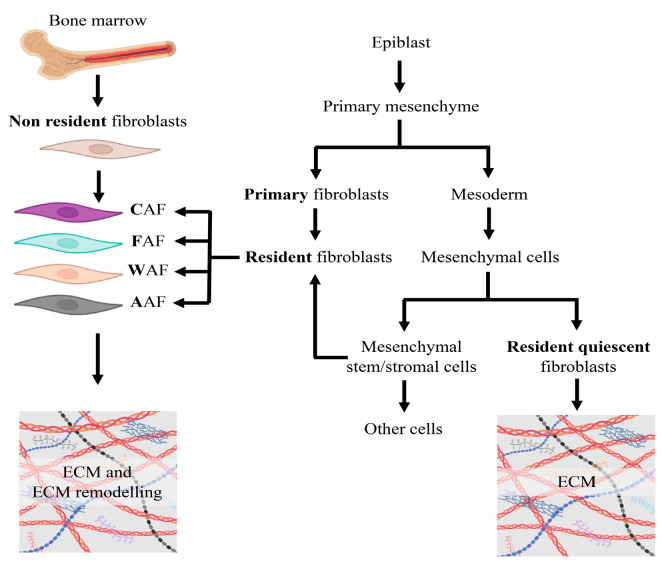
Developmental origin of fibroblasts. ECM, extracellular matrix; CAF, cancer-associated fibroblasts; FAF, fibrosis-associated fibroblasts; WAF, wound-associated fibroblasts; AAF, aging-associated fibroblasts.

Indeed, fibroblast heterogeneity already starts during development through evolutionary conserved mechanisms. Fibroblasts, known as “primary fibroblasts”, derive from the mesenchyme following epithelial mesenchymal transition of epiblasts, giving rise to “resident fibroblasts”. The primary mesenchyme is the source of the mesoderm from which originate different mesenchymal cell types, including fibroblasts that populate postembryonic developmental tissues. These cells, also described as “resident quiescent fibroblasts”, are the major contributor of extracellular matrix homeostasis, migrate, and respond to stimuli, exhibiting features of pluripotency. A subset of mesoderm-derived cells, known as mesenchymal stem/stromal cells (MSCs), are present in peripheral niches of adult tissues where they exhibit high manipulability, especially in terms of regeneration potential, and provide an additional supply of resident fibroblasts [9] (Figure 2). Beyond “resident quiescent fibroblasts”, also “non-resident fibroblasts” have been described, which may derive from direct conversion from one somatic cell type into another (e.g., bone marrow-derived mesenchymal stem cells or cellular trans-differentiation) (Figure 2). 

Fibroblasts from different body sites display distinct gene expression profiles (e.g., cell–cell signalling, matrix remodelling), highlighting the concept of “fibroblast topographical heterogeneity”. Therefore, age as well as the anatomical site of human dermal fibroblasts have been shown to affect functional differences, such as the ability to promote epidermal differentiation and to contribute to wound healing [10]. Indeed, dermal fibroblasts from the same topographic sites, even if isolated from different individuals, are more similar compared to those from different regions, although in the same subject. This “anatomical imprinting” is not modified by passages in cell culture, or by environmental changes [11]. Similarities and differences have been further highlighted by a seminal study by Chang and coworkers, who analysed the transcription profiling of fibroblasts from different sites and from different donors [12]. Whereas only a few genes were uniquely expressed by fibroblasts isolated from different sites, most of the genes showed a different expression grade depending on the fibroblast line. For instance, the expression levels of growth and differentiation molecules (e.g., members of transforming growth factor (TGF-*β*), Wnt, receptor tyrosine kinase and phosphatase families) were determined by the anatomic site (e.g., lung vs. skin). A similar trend was observed for extracellular matrix genes such as matrix metalloproteinase (MMP)-9, collagen type I and V, disintegrin and metalloproteinase domain-containing protein 12 (ADAM-12), ADAMTS like-1, perlecan, fibronectin 1, and fibrillin 1 [12].

Interestingly, it has been demonstrated that changes in the gene expression profile of fibroblasts depend not only on the organ (e.g., skin vs. lung vs. liver), but also on their position along the anterior/posterior, proximal/distal axes [11,13]. Indeed, during embryogenesis, site-specific cellular differentiation and tissue morphogenesis are associated with the expression of specific homeotic gene (*HOX*) [14]. For instance, *HOXD* gene expression is exclusively limited to dermal fibroblasts, thus suggesting that it is possible to determine the anatomic position of a fibroblast by looking at specific *HOX* gene expression [11]. Once established, the specific *HOX* gene expression is retained in the original pattern by “maintenance proteins” such as Polycomb-Group (PcG) and trithorax-Group (trxG) proteins acting as silencers or activators, respectively, of *HOX* and of many other genes [15]. The demonstration that these transcription factors specify site-specific transcriptional programs, conserved in adult cells and also after extensive ex vivo cell divisions [12], suggested the involvement of epigenetic mechanisms [16] or of noncoding RNAs [17]. Similarly to regulatory mechanisms observed in Drosophila [18], HOX proteins in adult fibroblasts modulate the expression of genes endowing fibroblasts with site-specific activities and inductive properties, although local influences that plastically alter fibroblast characteristics cannot be excluded [19]. 

The heterogeneity of fibroblasts may reflect the variety of cell markers that have been described in the literature. Although no universal fibroblast markers have been identified yet, some markers are considered “typical” of fibroblasts (e.g., ECM components such as vimentin or procollagen Iα2 chain; platelet-derived growth factor receptor alpha or CD34) [20,21], whereas others are more context-specific (e.g., discoidin domain receptor 2 (DDR2) in cardiac fibroblasts) [22]. According to the database CellMarker 2.0 (http://117.50.127.228/CellMarker/) (accessed on 6 June 2024), fibroblasts may express several mesenchymal markers which can differ depending on the tissue [23] (Table 1), and which are actually not unique, being shared with other cell types, including neurons, immune cells, epithelial cells, endothelial cells and adipocytes.

## 3. Fibroblasts and the Extracellular Environment 

Recent studies revealed the heterogeneity and the plasticity of fibroblasts dependent on the different skin areas, which have implications for cutaneous diseases and tissue engineering. As a matter of fact, early embryonic dermal fibroblast progenitors can potentially differentiate into several cell types, such as papillary or reticular fibroblasts, dermal papilla, and intradermal adipocytes [24]. Indeed, experimental studies have shown that, in vitro, papillary and reticular fibroblasts produce different amounts and types of ECM molecules, such as decorin and versican and similar amounts of biglycan [25,26].

The skin, depending on the areas, exhibits also differences in structural organization, balance between tensile strength and elasticity, and in the response to intrinsic and/or extrinsic stimuli. Therefore, fibroblasts, being the major producer of collagen and elastic fibres as well as of hundreds of molecules comprised within the so called “ground substance” [22], actively regulate tissue homeostasis and tissue repair [9]. Indeed, fibroblasts secrete and continuously remodel the ECM modulating the expression/activity of crosslinking enzymes (e.g., lysyl oxidase), of degrading enzymes (e.g., MMPs, tissue inhibitors of metalloproteinases) in response to specific stimuli and functional requirements [27]. To better understand the complexity of the ECM and the key role exerted by fibroblasts, a brief overview of the major groups of matrix molecules is provided.

### 3.1. Collagens

Collagens belong to a wide family of XXIX molecules characterized by a complex supramolecular structure exhibiting highly diverse morphologies across different tissues (Table 2).

**Figure 3:**
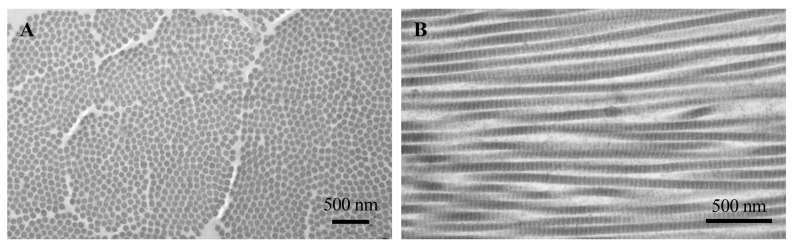
Transmission electron microscopy of collagen dermal fibrils cut transversely (**A**) and longitudinally (**B**). In longitudinal section, the fibrils show alternating light and dark bands. Images are from authors’ laboratory.

All members of this family exhibit (i) the amino acid repeating sequence [Gly–X–Y]_n_ with and without interruptions; (ii) the presence of proline and hydroxyproline in the X and Y positions of the typical basic sequence; and (iii) the supramolecular structural organization characterized by a right-handed triple helix formed from three left-handed polyproline α-chains of identical length, which gives collagen a unique quaternary structure. Collagen type I (approximately 70% of dry weight) and type III (8–11%) are the most represented in the adult skin with a ratio of collagen I:III of 4:1, in contrast to a 1:1 ratio in foetal and healing skin. Interestingly, the ratio between these two collagens regulates fibril width and stiffness, which are inversely related to the binding affinity of cells to the matrix. Indeed, hybrid molecules of collagen type I and type III, compared to fibrils made only of collagen type I or type III, are the most effective in terms of fibroblast activation, cell polarization and collagen synthesis [28]. Therefore, these fibrillar collagens (Figure 3) are of fundamental importance not only for their structural role in providing the required tensile strength to tissues, but also for triggering signalling events through the interactions with cell-surface receptors such as integrins, DDR, immunoglobulin receptors and leukocyte receptor complex [29].

Other minor collagens represented in the dermis are collagen type V, VI, VII, XII and XIV. Collagen type IV and XIX are mainly expressed in the basement membranes, whereas collagen type XIII, XVII and XXIX contribute to the epidermal strength [30]. Due to the their abundance, stability and biocompatibility properties, collagens, regardless of type and structure, are widely used as natural biomaterials to promote, for instance, wound healing, osteoblast migration and growth [31]. Collagen-based biomaterials have also attracted increased interest for their pharmaceutical potential for reconstructive and general surgery, cosmetology, drug delivery systems, tissue repair, and nutritional and therapeutic uses [32,33].

### 3.2. Elastic Fibres

**Figure 4:**
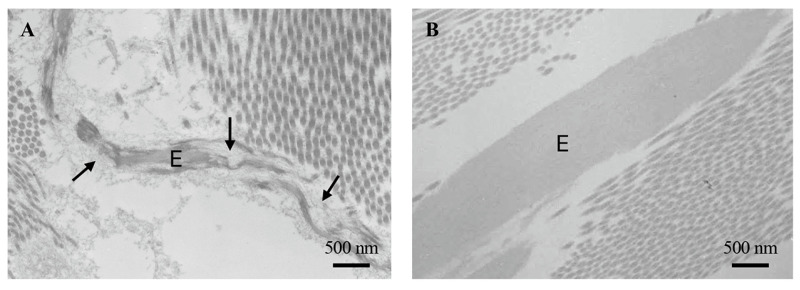
Transmission electron microscopy of elastic fibres in the skin of a healthy 5-day-old (**A**) and 50-year-old subject (**B**). The microfibrillar scaffold of elastic fibres is clearly evident in panel (**A**) (arrows). E: elastic fibres. Images are from authors’ laboratory.

Elastic fibres are typically present in higher vertebrates where they confer elasticity to soft connective tissues such as skin, vessels and lungs. The fibres are mainly composed of two components: the amorphous elastin and the microfibrillar scaffold, which are 10–12 nm wide filaments made of fibrillins, a group of glycoproteins enriched in Cys and representing the scaffold required to assemble mature functional elastic fibres (Figure 4) [34]. 

Inside the cells, the elastin precursor, tropoelastin, is transported to the membrane by the elastin binding protein (EBP), a 67 kDa protein that, binding to 61 and 55 kDa integral membrane proteins, forms a complex acting as the elastin receptor [35]. Outside the cells, due to the tropoelastin high hydrophobicity, it has been proposed that several other molecules may play a role in guiding tropoelastin monomers in the extracellular space favouring the assembly of the fibres, namely fibulins, glycosaminoglycans such as heparan sulphate, latent TGF-β binding proteins (LTBPs), ADAMTS (a disintegrin-like and metalloprotease- reprolysin type—with thrombospondin type I motif), microfibril-associated glycoproteins, minor collagens (e.g., collagen type VI, VIII, XVI, XVIII) [36,37]. In the skin, elastic fibres represent 2–4% of dermal dry weight, although their amount and distribution depend on the dermal areas, differing between subjects and with age [38]. Moreover, the reticular dermis contains thick, horizontally arranged elastic fibres with a prominent amorphous core, whereas the papillary dermis contains thinner perpendicular elastic fibres (elaunin fibres) connected with bundles of microfibrillar structures (oxytalan fibres) close to the dermal–epidermal junction [39]. Elastin secretion starts late in the foetus life, reaches very high levels during the neonatal stages, and then rapidly decreases after birth up to a point when elastogenesis is almost absent in adult life. Therefore, the extremely low turnover of elastin (approximately 70–75 years) makes this component the most resistant to stress and to damaging insults [40]. Nevertheless, several proteolytic enzymes can degrade elastin and/or its precursor tropoelastin (e.g., neutrophil and pancreatic elastases, cathepsins, MMP-2, 7, 9, 12 and 14) [41], leading to the formation of bioactive peptides called elastokines [42]. Interestingly, chemically synthesised or naturally derived elastin peptides, according to their specific sequences, can retain at least some properties of the whole molecules, thus suggesting that they can be used as bioinspired [43,44] or cosmetic biomaterials [45].

### 3.3. Glycosaminoglycans (GAGs) and Proteoglycans (PGs)

GAGs are complex polysaccharides ubiquitously expressed by mesenchymal cells. The different types of GAGs include heparin (HP), heparan sulphate (HS), chondroitin sulphate (CS), dermatan sulphate (DS), keratan sulphate (KS), and hyaluronic acid (HA) that, apart from HA, bind to core proteins, forming a wide spectrum of PGs. More than 50% of total HA in the body is present in the skin, either in the dermis (~0.5 mg/g wet weight) and in the epidermis (~0.1 mg/g) [46]. HA as well as other GAGs, due to their chemical structure and ability to interact with a wide range of cells and molecules, can be prepared and used in different formulations such as hydrogel, dermal filler, intradermal injection, scaffolds, creams, films, foams, and gels to regulate tissue water balance and to provide signals for different biological processes such as tissue regeneration, skin repair, cancer, wound healing, and inflammatory and immune responses [47]. Most GAGs bind a core protein (about 50 individual protein cores have been described), forming PGs, which are localized into the ECM, on the cell surface as well as intracellularly [48]. Versican, decorin, and biglycan, belonging to CS/DS-PGs, are highly expressed in the skin [49] and undergo dramatic changes in aging, photoaging and in cutaneous diseases [50]. As an example, KS-PGs, such as lumican, osteoglycin, and fibromodulin, play important roles in collagen fibrillogenesis, whereas HS-PGs, such as perlecan, endostatin, syndecans, and glypicans, are also expressed in the basement membrane, being essential for the survival and proliferation of keratinocytes [51]. 

### 3.4. Glycoproteins 

Within the complex extracellular environment, two glycoproteins are greatly involved in regulating signal transduction mechanisms through cell attachment and adhesion: laminin, typically localized on basement membranes, and fibronectin, abundantly found in both interstitial and basement membranes [52]. Laminins, in particular, are a large group of non-collagenous components of basement membranes at the interface between the epidermis and the dermis ensuring firm adhesion of the epidermis and protecting it against shearing forces [53]. Fibronectin has a different structure if it is secreted in the plasma or if synthesised by resident cells to form the extracellular network. After skin injury, fibronectin creates the so called “temporary fibronectin scaffold” serving as inducer of chemotaxis, angiogenesis and opsonisation of cell debris by inflammatory cells [54].

## 4. Fibroblasts, Mechano-Sensing, Mechano-Transduction and Mechano-Memory

Cells communicate with their environment, and the environment, in turn, may strongly influence cellular behaviour and cell fate, regulating tissue development and contributing to pathologic conditions [55]. Fibroblasts, for instance, “sense” the mechanical properties of the substrate (i.e., mechano-sensing) and transduce these signals (i.e., mechano-transduction) intracellularly, converting them into biochemical signals to modulate the cellular response, mainly through the “integrin adhesome” [56]. At the same time, cells can exert forces across the cell membrane against the underlying matrix through a continuous interplay that involves the cytoskeletal network and the transcriptional regulation [57]. Fibroblasts are known to respond to forces as well as to deformations exerted on the ECM. Therefore, during development, aging and/or diseases, changes occurring in the composition and in the extent of cross-links of matrix proteins (collagen, elastin, fibronectin, and matricellular proteins such as proteoglycans) regulate ECM topology and stiffness. Diverse mechanical cues can trigger distinct downstream signalling pathways, thus driving matrix stiffness-dependent cell migration [58]. Indeed, beyond chemotactic signals, the motility is also modulated by physical parameters, as clearly indicated by the process known as “durotaxis”, when fibroblasts migrate from soft to stiff substrates, being guided by the gradients of extracellular rigidity [59]. Indeed, mechanical stress, occurring for instance during the healing process, was observed already over 200 years ago, leading to the definition of the “ideal directionality of incisions” (i.e., Langer’s lines) to minimize the tension around the wound and consequently to limit scar formation [60,61]. Within this context, the “ECM-integrin-cytoskeleton-nucleus axis” has gained increased attention, hence promoting a better understanding of how cell extrinsic shear, tensile, or compressive forces regulate many aspects of development and pathology [62]. The rapid advances in the areas of molecular biology, biomechanics, and tissue engineering paved the way to the concept of “mechanotherapy” that is based on the employment of mechano-transduction mechanisms to stimulate tissue repair and remodelling [63]. Interestingly, it has been demonstrated that cultured fibroblasts, when grown on a soft substrate after a period on a stiff surface and *vice versa*, retain their previous behaviour at least for several days, indicating the existence of a “mechanical memory” [64]. 

Investigations on the biological and the biochemical role of the sustained effects exerted by mechanical stimuli have broadened the spectrum of possible mediators [65]. Evidence was provided indicating that the mechanical microenvironment, regulating membrane tension and disturbing the equilibrium between phospholipid molecules in the lipid bilayer, modulates, for instance, the activity of Piezo 1. This ion channel is a key mediator of the rigidity-dependent Ca^2+^ signalling [65], since it contributes to increase mitochondrial oxygen consumption rates and mitochondrial ATP production [66]. In turn, extracellular ATP modulates cell proliferation, migration, and differentiation, as well as contraction and relaxation [67]. Indeed, ATP is detected on cell membrane by P2Y purinergic receptors that favour the release of inositol 1,4,5-triphosphate (IP3) into the cytoplasm, the release of Ca^2+^ from the endoplasmic reticulum, and consequently, the diffusion of signalling molecules [66]. The regulation of ATP results from the balance between intracellular ATP (ranging from 1 to 3 mM) and extracellular ATP (ranging from 1 nM to 1 μM), which has a half-life of few seconds due to the activity of nucleotidases and of other hydrolytic enzymes, which degrade ATP, generating ADP, AMP and adenosine. ATP, at physiological pH, is largely anionic, and the concentration gradient favours the extrusion of ATP from the cell. Released ATP can initiate signalling pathways at concentrations that do not alter the intracellular energy stores [68]. Indeed, mechanical stretch can be intimately connected to ATP-related metabolism, calcium homeostasis, changes in integrin and cytoskeletal interactions [61]. It has been demonstrated that mitochondria activity as well as transcriptional and metabolic programs [69] can be modulated by the Hippo signalling pathway through the activation of the Yes-associated protein (YAP) and the transcriptional coactivator with PDZ-binding motif (TAZ) [70]. Indeed, nucleo-cytoplasmic shuttling of YAP/TAZ is strongly influenced by the composition of the ECM. Matrix stiffness, for instance, promotes the translocation of YAP/TAZ into the nucleus, leading to the activation of connective tissue growth factor transglutaminase-2, runt-related transcription factor 2 (RUNX2) and molecules related to the TGF-β signalling pathway, such as mothers against decapentaplegic homolog (Smad) -2, Smad-7, and p21. These molecules could be important for survival and regeneration, including dermal wound healing, for the development of fibroproliferative diseases and for cellular differentiation towards a pro-osteogenic phenotype [71,72,73]. On the contrary, miRNA-21, one of the most studied regulators of the pro-fibrotic transcriptional programme [74], was shown to inhibit the transcription of genes with anti-fibrotic actions, such as Smad7 and TGF-β receptor type III, and has been therefore proposed as a potential therapeutic target to control fibrosis and tissue repair [75].

Evidence has been provided from in situ and in vitro studies that disruption of mechano-signalling can alter fibroblast functions [76], and that matrix stiffness modulates chromatin accessibility, eventually regulating the epigenetic state and the transcriptional responsiveness of these cells to mechano-activation [77]. Indeed, mechanical cues can be remembered by cells for long- or short-term periods. While YAP/TAZ may act as a memory storage pathway on the shorter time scales, miRNA-21 may provide long-term storage of mechanical memory [78]. On the contrary, YAP/TAZ have a turnover rate of approximately 1–3 h and can shuttle in and out of the nucleus within minutes after exposure to different mechanical stimuli, thus indicating that these proteins regulate a short-term memory [78].

## 5. Fibroblasts and Skin Repair

**Figure 5:**
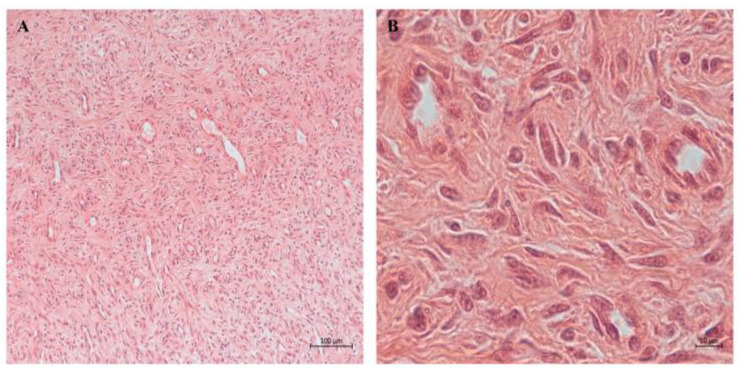
Light microscopy showing at low (**A**) and higher magnification (**B**) a human dermal granulation tissue sample characterized by several blood vessels and numerous elongated fibroblasts within a newly deposited extracellular matrix. Images are from authors’ laboratory.

Skin represents the first barrier against the external environment; therefore, any damage must be rapidly repaired, ensuring reconstitution of adequate structural resiliency. Wound repair is a complex, although well-orchestrated, process in which fibroblasts exert a major role, not only providing an exchange of signals between cells, but also secreting new ECM (Figure 5). Within this context, fibroblasts contribute to secretion and assembly of matrix components required for cell migration, provide signals for re-epithelialization, produce bioactive mediators promoting cellular differentiation, and regulate the immune response.

It is general assumed that fibroblasts involved in tissue repair originate from three different sources: resident fibroblasts that can be rapidly induced to proliferate, myofibroblasts, and circulating progenitor cells either differentiating into fibroblasts or endothelial cells [79,80]. Circulating fibroblasts (also known as fibrocytes) represent a group of bone marrow-derived mesenchymal progenitor cells migrating to sites of injury in response to cytokines and to matrix-derived chemokines. In the early 1960s, the names fibroblasts and fibrocytes were used to identify connective tissue cells in either the active or quiescent stage, respectively, as suggested by investigating the role of mesenchymal cells in wounding and healing processes [81]. Since then, interest has progressively grown over the role of fibrocytes also in pathologic conditions, and currently, there is a general, although not yet definite, consensus that cultured fibrocytes are long spindle-shaped cells with an oval nucleus which derive from peripheral blood monocytes and express several hematopoietic markers and low levels of collagen, whereas fibroblasts are stellate-shaped cells derived from connective tissues, producing ECM proteins and not expressing hematopoietic markers [82]. 

Although wound healing is always associated with scar tissue formation, it has been observed, already several decades ago, that foetus heals cutaneous injuries by regenerating a scarless dermal architecture [83]. This finding promoted countless studies aiming to identify the factor(s) capable of maintaining this regenerative potential during life. Indeed a complex interplay may contribute to the results of foetal wound healing, such as specific growth factor profiles (e.g., lower expression of bFGF (basic fibroblast growth factor), platelet-derived growth factor, TGF-β1 and 2 and higher expression of VEGF (vascular endothelial growth factor) and TGF-β 3 compared to postnatal healing), low inflammatory response, high content of collagen type III and hyaluronan in the newly deposited extracellular matrix, reduced biomechanical stress and the role of stem cells [84]. On the other hand, research on postnatal wound repair gained increased interest through the deepest understanding of fibroblast heterogeneity. In 2013, Driskell et al. showed that dermal fibroblasts arise from two different lineages [24]. The upper dermal lineage (i.e., papillary fibroblasts) is more involved in maintaining the epidermal structures, while the lower lineage (i.e., reticular fibroblasts) synthesises ECM, expresses higher amounts of α-smooth muscle actin and is largely responsible for dermal repair with the formation of scar tissue lacking hair follicles [85,86]. Interestingly, already in 2015, Rinkevich et al. described the presence of the ‘scarring fibroblast’ lineage associated with matrix deposition in the dorsal scar tissue in mice [87]. These cells were positive for both CD26 (also known as DPP4), a glycoprotein possibly involved in glucose metabolism, immunomodulation and tumorigenesis [88], and the homeobox protein engrailed-1 (EN1) that has been suggested to modulate inflammatory signals, survival and resistance to cell death [89]. More recently, Hu et al. found that the transcription factor paired related homeobox 1 (*Prrx1*) is activated and expressed in murine scar-producing fibroblasts in the ventral dermis [90]. These data further highlighted the importance of the areas of mesodermal origin of fibroblasts and suggested that the fibroblast origin may influence, for instance, the fibrotic behaviour in postnatal life. As a matter of fact, Wnt1 lineage-positive fibroblasts can contribute to scarless healing in specific contexts, such as the oral mucosa, whereas En1 or Prrx1 lineage-positive fibroblasts lead to scarring and healing in the dorsal and ventral skin, respectively [87,90]. Strengthening and stiffening of the ECM to form the mature scar takes place during the last stage of wound repair when fibroblasts continue to crosslink and replace some of the initially deposited collagen type III with collagen type I [91]. However, the altered balance of different matrix molecules may cause either chronic wounding or excessive scarring. Despite the countless studies performed in the last few decades, an area of significant unmet medical need is still represented by delayed wound healing that affects 1–2% of the population in countries of the developed world [92].

Several in vivo models, such as zebrafish or xenopus embryos, display regenerative healing [93] similar to that described in human embryos, where scar-free healing occurs until the 24 week of gestation [94]. Even though these animals represent interesting model organisms, mice and rats are, due to their well-known physiology, their manageable size and availability of adequate housing facilities, the most frequently used animal models for the study of different types of wound healing (e.g., excisional or incisional healing, ear and scalp wounds, chronic pressure ulcers, pathologic wounds, hypertrophic scarring). Nevertheless, they do not represent the best model for human wound repair because of their lower skin thickness (<25 μm vs. >100 μm of human samples), the abundance of hair follicles, loose skin, subcutaneous panniculus carnosus, and greater proportions of lymphocytes [95,96]. Interestingly, mouse wounds on the dorsal or ventral surfaces contract rapidly [97], thus resulting in a substantially smaller scar [96]. On the contrary, full-thickness wounds produced on the dorsal tail skin exhibit minimal contraction, causing a delay in wound closure, thus representing a suitable experimental model to investigate delayed healing processes [98]. In addition to the rodent wound healing model, rabbits and pigs are used to simulate acute or altered wounds such as in diabetic conditions or associated with malnutrition [99]. In particular, pig skin, due to its greater similarity to human skin in terms of structural characteristics and mechanical properties, is considered more appropriate than that of rodents to investigate wound healing for translational perspectives [100].

Although the various animal models have provided important insights into the process of wound repair in different experimental contexts, in vivo models are expensive and require adequate housing facilities, skilled personnel, and many animals to achieve statistically significant data. The 3Rs of animal experimentation (i.e., reduction, refinement and replacement) prompted researchers to intensify the search for alternatives to in vivo experimentation [101]. Several in vitro models have been developed over the years to evaluate cell proliferation and migration and test the efficacy of growth factors and/or of bioactive molecules for wound closure. In standard cell culture approaches, fibroblasts are grown on 2D surfaces, typically glass or plastic, with surfaces modified to favour cell adhesion. This approach has the advantages of obtaining a high number of cells for biochemical/biomolecular analyses and to visualize cells by imaging techniques [102]. In the scratch assay, for instance, a full-thickness scratch, introduced in the middle of the fibroblast monolayer, allows the quantification of the rate at which cells migrate and proliferate to close “the wound” [103]. However, 2D cell culture systems do not adequately mimic the tissue complexity; therefore, three-dimensional (3D) cell culture models were developed [104] (Table 3) to study cell–cell and cell–matrix interactions, investigate the paracrine effects of secreted molecules, and evaluate the amount and organization of newly deposited ECM [105,106]. 

In 3D matrix systems, to guarantee biocompatibility and non-immunogenicity, and with the aim to support, for instance, epidermis attachment, maintenance and stratification, to favour fibroblast migration and ECM deposition and to promote the blood vessels’ influx when implanted, different materials can be used. Natural components (e.g., gelatin, chitosan, fibronectin, hyaluronic acid, collagen, glycosaminoglycans, elastin, cellulose, alginate, silk, fibrinogen) or synthetic molecules (e.g., polylactic acid-PLA, polyglycolic -PGA, polyprolactine-PCL, polylactic-glycolic acid—PLGA) are utilised, either alone or in combination, based on their specific mechanical and biological properties [107,108]. Moreover, porosity, pore diameter and pore interconnection, depending on the methods used for crosslinking the polymeric structures (e.g., UV radiation, pH, temperature, chemical interactions), can further contribute to modulate the behaviour of fibroblasts and their ability to grow and secrete matrix components.

**Figure 6:**
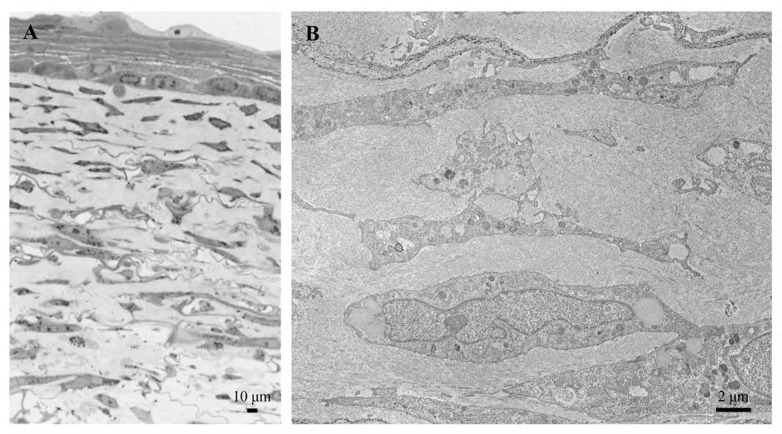
Skin equivalent observed by light microscopy (**A**). By transmission electron microscopy (**B**), numerous fibroblasts embedded within a newly deposited extracellular matrix are visible. Images are from authors’ laboratory.

The “skin equivalent” is a classic example of a 3D matrix system, in which fibroblasts are cultured on gelatin, on collagen, or on composite (e.g., chitosan plus gelatin plus hyaluronic acid) scaffolds until the formation of a “dermis equivalent” (e.g., fibroblasts embedded in ECM) [109,110]. Then, keratinocytes are seeded on the top of the dermis, and after approximately 2–3 weeks, the layer of keratinocytes is in place at the air–liquid interface to promote stratification of the epidermis (Figure 6).

It has been shown that the skin equivalents, in terms of proliferation, migration, growth-factor responsiveness and protease expression, better reproduce the in vivo environment [111], although this model lacks fundamental processes for wound healing, such as inflammation and angiogenesis [112]. Skin equivalents can be produced in the laboratory by skilled personnel, but the complexity of the system and the need to ensure reproducible conditions and environmental features have prompted the development of automated systems suitable for the generation of complex tissue architectures [113]. 

Three-dimensional bioprinting is a promising and rapidly expanding technology that better allows the creation of three-dimensional structures, in a layer-by-layer manner, in which cells and biomolecules are added to defined positions, forming constructs closely mimicking native tissue and organ architecture [114,115] (Table 3). Using a wide range of natural or synthetic polymers, extracellular proteins, and minerals, bioprinting was demonstrated to efficiently support both cell viability and proliferation [116]. In fact, 3D bioprinting can produce custom-made skin grafts that match the patient’s wound geometry, improving integration and healing rates. Indeed, several studies have shown that bioprinted skin constructs can enhance re-epithelialization, angiogenesis, and collagen deposition [115,117]. Interestingly, in recent years, 3D has evolved into 4D/5D/6D systems. ”4D” refers to the time dimension, signifying that the product changes shape after printing due to the stimulus; “5D” implies the ability to create concave or curved shapes to better maintain cellular and mechanical properties. With the 5D technology, in particular, it seems feasible to integrate machine learning and artificial intelligence to generate smart materials for multifunctional, ecological, and biocompatible components [118]. For instance, bio-patches can be made to identify specific infections through design or to deliver drugs. Finally, the “6D” identifies the ability of the material to modify its shape and/or exhibit intelligent behaviour with unique stimulus-response features [119], such as in vascularized tissue scaffolds [120]. 

It is well known that the wound healing environment is modulated by several growth factors locally produced by mesenchymal and inflammatory cells and that their exogenous administration can counteract delayed and/or impaired wound healing, such as that observed in aged or diabetic patients [121]. However, exogenous application of growth factors has demonstrated lower efficacy than expected due to limited in vivo stability, restricted absorption through skin around wound lesions, and elimination by exudation prior to reaching the wound area [122]. Currently, several in vitro and in vivo studies are investigating the role of extracellular vesicles (EVs) derived from mesenchymal stem cells or from platelets to enhance skin wound repair [123,124,125,126]. This approach may be rather challenging and requires defining and standardising proper sources, extraction techniques, and storage conditions. Recent studies have demonstrated that platelet-derived extracellular vesicles (pEVs), providing growth factors and signalling molecules, can enhance endothelial cell angiogenic potential, as well as also the proliferation rate and the migration capabilities of dermal fibroblasts. These data supported the first in-human, double-blind, placebo-controlled, phase I clinical trial on healthy volunteer adults. Result paved the way to further explore the efficacy of using pEVs in future clinical trials for treating delayed wound healing [127].

The successful use of platelet-rich plasma for the treatment of chronic skin wounds or diabetic foot ulcers further underlines the benefits of platelet-derived molecules in modulating innate and adaptive immunity and inflammation, as well as wound-healing and tissue-repair mechanisms [128]. Within this context, it is also important to understand the complex interactions between skin-resident fibroblasts and immune cells [129]. For instance, in the perinatal period, CD4+ regulatory T cells (Tregs) home into the skin where they interact with collagen type I through integrin alpha 2 and with hyaluronan through CD44 to mediate immunotolerance. Therefore, matrix components secreted by dermal fibroblasts create stromal niches influencing the migration of antigen-presenting cells, such as Langerhans cells, which migrate from the epidermis to the dermis by interacting with laminins within basement membranes through integrin alpha 6. The multifaceted immunomodulatory functions of fibroblasts have been highlighted in the recent years, demonstrating that they respond to stimuli from inflammatory cells and/or provide signals to immune cells [130]. Fibroblasts exert their immune regulatory functions mainly via their inflammatory secretome (e.g., chemokine ligands, interleukins, nitric oxide, prostaglandin, TGF-β) acting in synergy with angiogenic remodelling growth factors such as VEGF and with MMPs and their inhibitors [131]. Moreover, fibroblasts, depending on their tissue origin, can either promote or inhibit the recruitment of leukocytes by modulating the cytokine-induced expression of adhesion molecules on endothelial cells, and can also regulate leukocyte behaviour and survival within damaged tissue through a cross talk mediated by the surface antigen CD40 [132] and toll-like receptors (TLRs) [133]. In addition to their role in the innate immune response, fibroblasts can also interact with both B and T lymphocytes, contributing to regulate their activation [134], possibly avoiding unnecessary immune response. The ability of fibroblasts to act as antigen-presenting cells and to be reprogrammed into functional dendritic cells may play an important role in cancer biology, being able to present antigens to T cells, promote the immune response and possibly influence anti-tumour immune therapies [135,136].

## 6. Fibroblasts and Skin Aging

Aging is characterized by a progressive functional decline of organs and tissues and associated with a 35% reduction in active fibroblasts in old individuals [137,138], together with an increase in senescent cells. It was in the early 1960s that Hayflick first introduced the concept of replicative senescence to indicate that normal cultured human fibroblasts display a finite capacity for cell division [139]. Actually, senescence occurs throughout the lifespan when a proliferating cell undergoes a stable cell cycle arrest, becoming resistant to growth-promoting stimuli, typically in response to DNA damage [140]. Therefore, senescence can be considered either a protective mechanism blocking the proliferation of damaged cells, or a deleterious process contributing to age-related disorders, including cancer, neurodegeneration, and metabolic and cardiovascular diseases [141].

The so-called senescence-associated secretory phenotype (SASP) includes a mixture of molecules such as cytokines, MMPs, miRNAs, chemokines, growth factors, and small-molecule metabolites that, when released by senescent cells, can exert immunoregulatory effects and affect the proliferation and motility also of non-senescent cells [142]. Due to its paracrine and autocrine effects, SASP may contribute to cutaneous aging, such as reduced thickness, loss of elasticity, progressively increased wrinkles and defective and/or delayed wound healing [138]. Within this context, it is worth mentioning that a vicious loop exists between oxidative stress (i.e., unbalanced ratio between reactive oxygen/nitrogen species—ROS/RNS—and antioxidant systems) and inflammation. ROS/RNS serve as signalling molecules triggering inflammatory responses, but inflammatory cytokines and chemokines, in turn, generate more ROS/RNS active on several pathways, including mitogen-activated protein kinase (MAPK), nuclear factor kappa-light-chain-enhancer of activated B cells (NF-κB), TGF-β, and mechanistic target of rapamycin (mTOR), leading to the accumulation of senescent fibroblasts [143]. As a matter of fact, senescent cells play a critical role in the development of aging-related changes; therefore, it has been proposed that their elimination may arrest or even reverse the process. 

Senotherapy is an experimental approach that, through senolysis (i.e., destruction of senescent cells) and senomorphic therapy (i.e., reversal of senescence-related changes), aims to reduce the number of senescent cells and, consequently, delay and/or reverse, to some extent, aging [144]. Among the agents that have been tested for their ability to eliminate senescent cells, metformin is one of the most promising, as suggested by the Targeting Aging with MEtformin (TAME) study, a randomized controlled study performed on 3000 patients aged 65–79 without glucose intolerance [145]. More recently, Pep 14 was demonstrated to be a promising senomorphic molecule since, through the activation of a serine/threonine phosphatase (PP2A), it decreased the level of senescence markers also in fibroblasts from a premature aging syndrome (i.e., Hutchinson–Gilford Progeria Syndrome). Moreover, in 2D and 3D aged skin models, this peptide improved numerous skin health markers [146].

**Figure 7:**
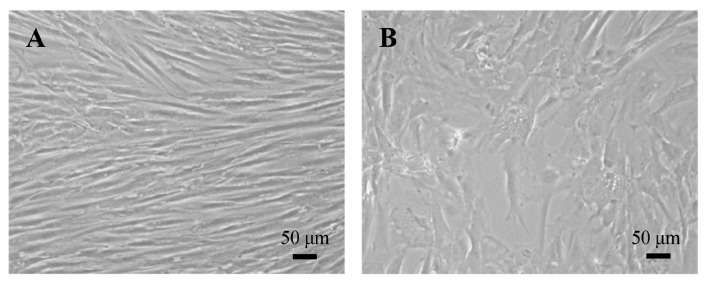
Light microscopy of human dermal fibroblasts at low (**A**) and high (**B**) cumulative population doubling. When cells reach replicative senescence, fibroblasts become larger and accumulate cytoplasmic vacuoles (**B**). Images are from authors’ laboratory.

Dermal fibroblasts, similarly to many other senescent cells, are characterized by morphological changes (e.g., irregularly enlarged and flattened shape) (Figure 7), metabolic adaptations (e.g., mitochondrial dysfunction, altered redox balance and enhanced lysosomal activity), chromatin reorganization associated with a DNA damage response, and altered signalling pathways such as MAPK, NF-κB, TGF-β, and mTOR. Moreover, modified gene expression and secretory phenotype lead to cytoplasmic accumulation of lipid residues (i.e., lipofuscin), secretion of MMPs, release of metabolites and small molecules with immunomodulatory effects, as well as extrusion of extracellular vesicles that contain high levels of IL-6 and are less effective on keratinocyte differentiation [138,147].

The altered balance between synthesis and degradation of ECM components observed in dermal aging is maintained in vitro in human dermal fibroblasts [148], further supporting the use of these cells to investigate the aging process and to test anti-aging treatments. An interesting study was performed comparing metabolic and synthetic parameters in human dermal fibroblasts isolated from young and old subjects (ex vivo aging model) and cultured from early up to late cumulative population doublings (in vitro aging model). Data clearly demonstrated that some changes occur in vivo, possibly induced by the aged environment, and are maintained in vitro, whereas other changes take place in vitro, being associated with progressive cell doublings and cell senescence. In particular, fibroblasts from aged donors exhibit impaired redox balance independently from population doublings, whereas proteins related to heat shock response, or involved in endoplasmic reticulum and membrane trafficking, appeared differentially expressed only during in vitro aging, indicating that in approaching senescence, the whole cell machinery becomes permanently altered [149]. For instance, during aging, soft connective tissues (e.g., skin and blood vessels) appear more prone to developing ectopic calcification, and an in vitro study has demonstrated that neonatal dermal fibroblasts, at both low and high passages, are less responsive to pro-calcifying stimuli compared to fibroblasts isolated from adult donors [150], once again suggesting that aging fibroblasts can lose their specialized identity, leading to altered functions, such as an increased expression of osteogenic genes [151]. Similar results were obtained in the Genetic and Epigenetic Signatures of Translational Aging Laboratory Testing (GESTALT) study, where proteins extracted from fibroblasts isolated from skin biopsies from 82 persons (22 to 89 years old) underwent liquid chromatography-mass spectrometry analysis. Among the proteins differentially expressed as a function of donor age, two pathways appeared mainly involved in older individuals (i.e., autophagy and antioxidant defence), whereas two pathways were less represented (i.e., ribosome biogenesis and DNA replication and repair) [152]. In agreement with these data, both mitochondrial function and integrity become impaired with aging [153,154] and not only in skin areas exposed to UV radiation that are known to induce photoaged-changes [155]. Dysfunctional mitochondria may in fact produce ROS at a level that exceed the buffering capacity of the antioxidant system. Aberrant redox balance, in turn, promotes cell cycle arrest and premature senescence [156], affecting mitochondrial respiration through the dysregulation of glycolytic enzymes [157]. It is well known that dermal aging is typically characterized by loss of elasticity [38]. ROS and RNS favour post-translational modifications such as glycation, nitrosylation and carbamylation, also affecting the mechanical behaviour of the elastic component and contributing to the degradation of elastic fibres. These fibres, especially in pathologic conditions, can become more susceptible to damaging environmental stimuli, variably interact with other matrix components or ion species which accumulate inside the fibres, and may release bioactive peptides [40].

## 7. Fibroblasts and Diseases

As fibroblasts can be easily isolated from small skin samples and extensively cultured in vitro [158], these cells represent one of the best known reductionist approaches to investigate heritable disorders, test responses to drugs, and dissect signalling pathways and/or metabolic signatures in a broad spectrum of physio-pathologic conditions ranging from aging, wound healing, and connective tissue diseases to metabolic disorders and even psychiatric pathologies [149,159,160]. 

Indeed, fibroblasts have been widely used to investigate the biochemical defects associated with heritable connective tissue diseases characterized either by (i) defective synthesis of matrix components, such as *Cutis laxa* [161], Ehlers–Danlos and Osteogenesis imperfecta syndromes [162]; (ii) excessive production/accumulation of matrix molecules such as mucopolysaccharidoses [163] or altered cellular behaviour such as in fibrotic diseases [164,165,166,167]; (iii) autoimmune diseases [165,168]; or (iv) ectopic calcification [169,170,171,172,173,174,175]. Moreover, the anatomic diversity of human skin is also mirrored in the site specificity observed in many cutaneous lesions. These differences raise the question of how cells are capable of maintaining their identity despite the complexity of self-renewing tissues in which they are located [13]. 

The functional heterogeneity and unique plasticity of fibroblast origin may operate through epigenetic memory [176] associated with distinct DNA methylation patterns. Epigenetically modified genes modulate fibroblast proliferation and response to exogenous stimuli. For instance, aberrant methylation of specific promoters leads to transcriptional silencing of genes such as *RASAL1* (RAS protein activator-1), *FLI1* (Fli-1 Proto-Oncogene, ETS Transcription Factor) and *PPARγ* (Peroxisome Proliferator Activated Receptor Gamma). Interestingly, PPAR*γ* hypomethylation was observed with liver fibrosis [177]. DNA methylation may also be a consequence of altered redox balance. Indeed, ROS can be associated with either global DNA hypomethylation or histone modifications leading to oxidation of basic amino acids such as arginine and lysine, which may affect chromatin relaxation and accumulation of several transcription factors [178]. Indeed, epigenetic modifications have also been extensively investigated in recent years to better understand the interaction between cancer cells and the surrounding stroma.

Cancer-associated fibroblasts (CAFs) play a key role in cancer progression and metastasis. They derive from various cellular sources, including reprogrammed resident fibroblasts [179], bone marrow-derived mesenchymal cells [180], adipocytes [181], endothelial cells through endothelial–mesenchymal transition [182], and epithelial cells through epithelial–mesenchymal transition [183]. The recent description of “cancer as a wound that does not heal”, although restricted to some aspects of carcinogenesis, nevertheless clearly underlines the central involvement of fibroblasts [184]. Indeed, CAFs recruit immune cells and activate angiogenesis by secreting a variety of growth factors (e.g., TGF-β and VEGF), cytokines (e.g., IL-6), chemokines, extracellular vesicles, and extracellular matrix components (e.g., collagen and MMPs) that play a crucial role in tumour growth [183,185]. As an example, TGF-β is secreted by CAFs and plays a crucial role in the crosstalk between stromal and cancer cells, inducing epithelial–mesenchymal transition and metastasis through the activation of the transcription of HOX transcript antisense RNA (HOTAIR) [183]. Another soluble factor produced by CAFs is FGF, which promotes, in turn, CAF expansion through transcriptional repression of p53 in skin [186]. Interestingly, FGF and TGF-β, during tumour development, modulate different processes, inducing inflammation and epithelial–mesenchymal transition/invasion, respectively [187]. These findings further underline the complexity of the cancerous environment, but also the importance of the stroma in modulating tumour expansion. Using single-cell RNA sequencing and spatial proteomics data, it was demonstrated that, within the tumour microenvironment, there are different CAF subpopulations characterized by distinct phenotypes and functions which, depending on the stimulus (i.e., molecules produced, hypoxia, ROS levels), can greatly influence tumour development [188,189,190]. It has been recently suggested that different subtypes of CAF can predict skin cancer malignancy [191]. Immuno CAFs (iCAFs) express proinflammatory cytokines (i.e., IL-1β, IL-6) and chemokines, whereas matrix CAFs (mCAFs) exhibit increased matrix production. These two cell types seem to be mutually exclusive and possibly associated with skin cancers with higher (iCAFs) and lower (mCAFs) metastatic potential. In the tumoral environment, ECM remodelling induces biomechanical and biochemical changes favouring cancer cell survival, invasion and metastasis [192]. Indeed, solid tumours contain large ECM deposits that can constitute up to 60% of the tumour mass, the great part being composed of molecules belonging to the collagen family. Altered ratios between collagen types, increased proteolytic activities (e.g., MMP and ADAMs), post-translational modifications, such as changes in proline and lysine hydroxylation and lysyl-oxidase dependent crosslinks, further support the role of CAF and the ECM in conditioning tumour progression and migration, thus paving the way for new therapeutical targets [193]. In addition to collagen(s), a key role in regulating proliferation, adhesion and migration is exerted by proteoglycans that can regulate ECM assembly, cellular signalling and mechanobiology in both normal and transformed tissues. Perlecan, for instance, is significantly upregulated in CAF, thus creating an environment permissive to cell invasion and metastasis [194]. By contrast, decorin, a small leucine proteoglycan, acts as a versatile tumour suppressor, acting on autophagy, mitophagy, angiogenesis, apoptosis, and cell cycle-arrest, thus providing a potential strategy for cancer therapy [195]. Moreover, decorin has the ability to sequester the cytokine TGF-β, thereby diminishing its activity [196]. Therefore, it has become increasingly evident that future therapeutic approaches will need to consider the heterogeneity and functional complexity of CAFs and of their stroma to effectively treat a specific tumour.

## 8. Dermal Fibroblasts by High-Throughput Technologies

Given the role of fibroblasts in providing the homeostasis of connective tissues in which they are embedded, and their spreading use as experimental models, high-throughput approaches provide highly informative tools to better characterize transcriptional and protein profiles. 

Analysis of the transcriptome profile of skin fibroblasts demonstrated that these cells maintain proliferative, secretory activity and genomic stability during culture in subsequent passages, at least up to the tenth passage [197,198], in accordance with the seminal study by Hayflick and Moorhead, showing maintenance of the genomic stability of human fibroblasts even after 40 generations [139]. The family of genes mainly involved in the characterization of skin fibroblasts belongs to the following GO groups: cell adhesion, extracellular matrix organization, and collagen fibril organization [198]. Recently, transcriptome analysis of RNA from skin fibroblasts was revealed to be a powerful technique for the detection of pathogenic splice variants in genetic diseases (e.g., deep intronic changes, transposable element insertions and postzygotic pathogenic variants) that are missed by routine DNA sequencing [199]. Furthermore, in psoriatic patients, a combined transcriptome analysis of skin fibroblasts and of whole blood, followed by analysis of their interactome, revealed differentially expressed genes related to platelet activation and hippo signalling pathway capable of discriminating between responders and non-responders as early as one month after treatment [200]. These findings indicate that investigating the transcriptome of skin fibroblasts may have diagnostic and prognostic potential in many pathologic conditions.

**Figure 8:**
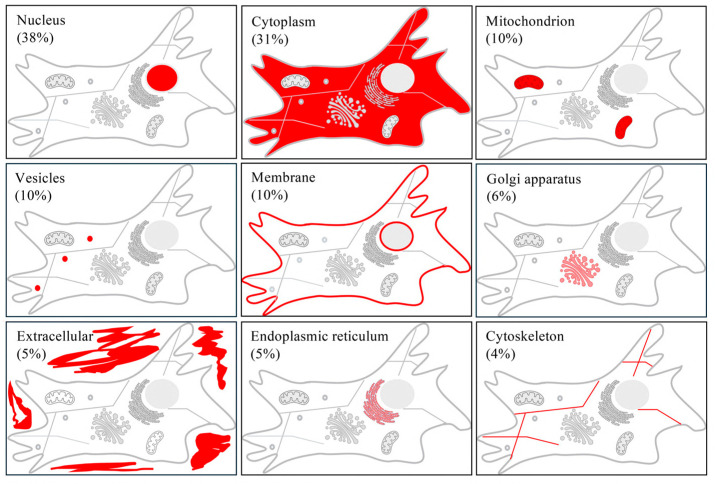
Proteomic analysis of cultured human dermal fibroblasts allows the identification of proteins belonging to different subcellular compartments. Some proteins are exclusively present in one compartment, whereas others are detected in different compartments.

The Human Genome Project identified about 40,000 genes that are translated into approximately 300,000 to 1 million proteins considering alternative splicing and post-translational modifications. However, proteins exhibit different sizes and chemical characteristics thus making their isolation and identification more challenging. Nevertheless, proteome analyses provide an extraordinary source of data to evaluate and understand how cells behave and contribute to physiological or pathological conditions, respond to exogenous stimuli, and undergo changes in protein interactions [201]. For several years, gel electrophoresis, in particular 2D-PAGE, combined with image analysis and mass spectrometry (MS) identification, has been widely applied in different experimental conditions [153,202,203,204], since it is capable, based on the isoelectric point and the molecular weight, of separating complex protein mixtures into single protein spots. However, 2D-gel based proteomics is laborious and time-consuming, and it is unable to separate proteins that are too large, too small, too acidic or too basic [205]. Within this context, a crucial step is represented by sample preparation [206]. Therefore, several extraction and fractionation methods have been developed to reduce time and improve reproducibility, thus increasing throughput, especially in the context of connective tissue complexity [207,208]. Moreover, in recent decades, “shotgun proteomics” has been developed and spread [209]. The shotgun approach refers to the analysis of complex protein mixtures that are digested into peptides and then separated by liquid chromatographic tandem mass spectrometry (LC-MS/MS), allowing the identification and quantification of thousands of proteins. In particular, from whole cell protein extraction, approximately 5000 proteins can be identified by LC-MS/MS (Figure 8), although this number may increase significantly when analysing peptides fractionated using high-pH reverse phase-LC fractionation [152]. 

Although extracellular proteins can be identified among those in a cell lysate, a more precise understanding of the extracellular environment in terms of matrix, matrix-associated proteins (e.g., different collagen types, proteoglycans, metalloproteinases, protease inhibitors) and extracellular vesicles [172,210] requires analysis of the secretome, i.e., proteins released from the cell into the extracellular space. Studying the secretome remains challenging despite the development of various strategies/methodologies for protein isolation [211]. Nevertheless, several studies have analysed the fibroblast secretome under different physio-pathological conditions, for example, in wound healing [212], in ectopic calcification [172] and in skin cancer [213]. Cell cultures are valuable tools for investigating the biology, biochemistry, physiology and metabolism of cells; however, the in vitro environment significantly differs from the natural environment of the human body in which cells reside. Interestingly, tissue imaging proteomics or imaging mass spectrometry are emerging tools for identifying and mapping the spatial distribution of proteins across a tissue without the removal of cells or homogenization of tissue [214]. This label-free analytical technique has been applied, for instance, to analyse the fibroblasts in their natural microenvironment and to characterize the extracellular matrix [215,216].

Therefore, transcriptomics and proteomics represent fundamental and complementary techniques for better investigating and modulating signalling pathways, improving the delivery of cells and/or molecules in situ (e.g., smart matrix technology), limiting undesired effects such as inflammation and fibrosis, and better adapting to tissue specificities and patient’s conditions [217]. These approaches will have a fundamental impact in the context of the future development of tissue bioengineering and of regenerative medicine. The fascinating and widespread area of regenerative medicine has been the subject of an exponentially increased number of studies in the last two decades better disclosing the nature and potential of stem cells [218]. Several regenerative medicine-based approaches have been recently described also for skin regeneration, such as the use of mesenchymal stem cells (MSC), tissue induced pluripotent stem cells (iPSCs), fibroblast-based products, and blood-derived components as well as extracellular vesicles/exosomes [219]. However, their complexity is far beyond the scope of this review, and we invite the readers to refer to more-specific literature. Meanwhile, tissue engineering has attracted growing interest at the forefront of biomedical research, with great expectations for personalized healthcare and regenerative medicine. Integrated in vitro systems have, in fact, expanded the possibilities for disclosing important cues in the understanding of tissue biology, and also for replacing diseased and/or damaged tissues [220,221,222]. In the previous section, we described the major characteristics of different substrates and scaffolds underlining the importance of cell–matrix interactions. However, despite the large number of bioengineered skin substitutes available on the market, the improved knowledge of fibroblast behaviour in physio-pathological conditions, and the expanding role of the extracellular matrix as a structural support and a source of signalling molecules, research is still urgently needed to improve the standardization of production processes and storage as well as the biomechanical and biological performance of matrices/substrates to closely mimic tissue architecture and function.

## 9. Conclusions

Fibroblasts are responsible for the synthesis, deposition and maintenance of soft connective tissues. Changes in composition and organization of the extracellular matrix modulate the behaviour of these cells that activate specific signalling pathways sensing the biomechanical properties of the surrounding environment. Despite their developmental heterogeneity, fibroblasts, since they can be easily isolated from tissues and grown in culture on artificial surfaces or on natural and/or bioengineered materials, are and will be one of the most widely used model systems for understanding skin aging and repair, for investigating pathologic conditions through reductionist approaches, and for applications in biomedicine.

## Figures and Tables

**Table 1:** Fibroblast markers according to the database CellMarker 2.0. Data were filtered for human tissue, normal cell and fibroblast.

Tissue	Marker
Adipose tissue	CD45, CD91
Articular cartilage	COL1A1, COL3A1, COL5A1, S100A4
Bile duct	FAP, PDGFR-b
Bladder	COL1A1, COL1A2, COL3A1, DCN, PLA2G2A, S100A4
Blood vessel	COL1A1, COL3A1, DCN, PDGFRb
Brain	BRACHYURY, COL1A1, DCN, IGFBP5, KCNT2, LGALS3, PDGFRA, SNAI1, THY1
Breast	CK18, CK19, CK8, POSTN
Capillary	ACTA2, MYH11
Cardiac atrium	ADAMTS4, AXL, VCAN
Dermis	CD34, CAM1, IL6, PDPN
Esophagus	ACTA2, COL1A1, COL3A1, CTGF, PDGFRa, PDGFRb, PLN, SERPINE1, TAGLN, VIM, WNT2B
Eye	MGP, MYOC
Eye choroid	COL1A1, FBLN1
Heart	ACTA2, ALDH1A1, CADH2, CKAP4, COL1A1, COL2A1, COL3A1, DCN, DDR2, FN1, ITA4, LTBP2, PDGFRA, POSTN, TGF-21, THY1
Intestine	C7, COL1, POSTN, ALKAL2, BMP2, BMP3, CCL21, CD24, CENPF, CLEC2B, COL28A1, COL6A5, COL9A3, CXCL10, CXCL13, DLK1, EBF2, EBF3, FBLN1, GDF10, GINS2, GPX3, GREM1, HAND1, HMGA2, IFIT3, INSC, LXN, MBP, MKI67, MMP11, NPY, NR2F1, OLFM3, OSR2, PCLAF, PDLIM3, POSTN, PRRX1, PTGDS, PTN, RAMP1, SCN7A, SCUBE2, TFPI2, THY1, TNFSF11, TOP2A, THYMS, WNT4, WNT5B, CD90
Kidney	COL1A1, COL3A1, DCN, DDR2, FSP1, LUM, MYL12B, SPP1, VIM
Limb skin	CCL19, CCL2, COL18A1, COL6A5, DCN, TNC
Liver	FSP-1, NDUFA4L2, RGS5, S100A4
Lung	COL1A1, COL3A1, PDGFRa, CC2512, alpha-SMA, ACTA2, CD34, CD36, CD90, CD97, CDKN1A, CTGF, DDR2, Desmin, DKK3, FBN1, GAS6, GSN, KMT2B, LUN, MIF, MYC, OSR1, P4HA1, PDGFRB, PDGFRA, PLIN2, PRRX1, S100A4, SNAI1, TCF21, VCAN, VIM, VIT
Muscle	MYH1, MYH7,
Myocardium	TGF-β1
Ovary	BGN, COL1A1, COL1A2, DCN, POSTN
Oviduct	DCN
Pancreas	ACTA2, COL1A1, COL1A4, SPARC
Periodontium	COL1A1, DCN
Peritoneum	COL1A2, LUM, MMP2, DPGFR
Prostate	APOD
Skin	APCDD1, APOD, APOE, CCL19, CD26, CD36, CD90, CFD, COL1A1, COL1A2, COL2A2, COL6A2, COL6A5, CXCL12, CXCL14, DCN, FAP, FB, FBLN1, FN1, KRT14, LUM, LYVE1, MEG3, MMP2, MXRA8, PAX1, PCOLCE, PDGFRA, PLA2G2A, PLPP3, SDC2, SERPINH1, SFPR4, SMA, TRIL, VIM
Spleen	C11orf96, CALD1, COL4A1, CXCL13, IGFBP2, IGFBP7, PTGDS, SPARCL1, TAGLN, TIMP1
Stomach	POSTN, SMA
Synovium	COL3A1, VIM, CD55, GGT5, CD90, ISLR, PDGFRA, PDPN,
Thymus	ALDH1A2, COLEC11, FAP, FBN1, GDF10, PDGFRA, PI16, S100A4, SEMA3D
Uterus	SFRP2, APOD
Vagina	FBLN1, LUM
Vocal fold	CD105, Cd14, CD29, CD31, CD34, CD44, CD45, CD73, CD90
Undefined	α-SMA, BGN, CD10, CD105, CD11b, CD121a, CD140a, CD140b, Cd19, CD29, CD34, Cd44, CD45, CD47, CD73, CD81, CD90, CD91, COL1A1, DDR2, ECM2, FBLN1, HLA-DR, MFAP5, PDGFRA, PDGFRB, S100A4, TGF-β1, VEGF, vimentin

**Table 2:** Collagen types can be divided according to their supramolecular organization (in bold are the collagens localized in the skin). MACIT, membrane-associated collagens with interrupted triple helices; FACIT, fibril-associated collagens with interrupted triple helices; MULTIPLEXINS, multiple triple-helix domains and interruptions.

Collagen Family					
Fibril Forming Collagens	Network Forming Collagens	MACIT Collagens	FACIT Collagens	MULTIPLEXINs Collagens	Other Collagens
**I** II **III** **V** XI XXIV XXVII	**IV** **VI** **VII** VIII X	**XIII** **XVII** XXIII XXV	IX **XII** **XIV** XVI **XIX** **XX** XXI XXII	XV XVIII	XXVI XXVIII XXIX

**Table 3:** Comparison of in vitro models showing advantages and disadvantages.

Model	Advantages	Disadvantages
**2D cell culture**	- Fast proliferation - Easy culture set-up - Very good reproducibility - Suitable for high throughput - Relatively low cost	- Monolayer system - Absent concentration gradient - Different cellular morphology and molecular mechanisms vs. in vivo - Limited cell–matrix interactions
**3D matrix cell culture**	- Better mimicking of tissue stiffness - Good cell–matrix interactions - Possibility of co-culture systems - Presence of concentration gradients - Similar morphological and physiological characteristics compared to in vivo	- Complex culture set-up - Most models do not fully reconstitute all cell–cell and cell–matrix interactions, or functionality, of the skin - Skilled personnel are required - Expensive
**3D bioprinting**	- Custom-made architecture - Multiple co-culture possibilities - Better reproduction of tissue heterogeneity - High-throughput production	- Limited choice of materials which depend on the bioprinting system - More expensive

## Data Availability

Data sharing is not applicable to this article.
